# Gypenosides Mitigate STEC-Induced Intestinal Injury Through Modulation of Ferroptosis Involving the ATF4-CHAC1-GPX4 Axis

**DOI:** 10.3390/antiox15091103

**Published:** 2026-09-01

**Authors:** Fei Su, Liwen Liang, Bin Yu, Lihua Xu, Hongchao Sun, Shiyi Ye, Xiufang Yuan, Yin Xue, Canying Liu, Junxing Li

**Affiliations:** 1 Institute of Animal Husbandry and Veterinary Science, Zhejiang Academy of Agricultural Sciences, Hangzhou 310002, China; sufei@zaas.ac.cn (F.S.); liang_liwen@gzlab.ac.cn (L.L.); yub@zaas.ac.cn (B.Y.); xulihua@zaas.ac.cn (L.X.); sunhongchao@zaas.ac.cn (H.S.); yesy@zaas.ac.cn (S.Y.); yuanxf@zaas.ac.cn (X.Y.); 2 College of Animal Science and Technology, Foshan University, Foshan 528225, China; liucy3032@fosu.edu.cn; 3 Zhejiang Center of Animal Disease Control, Hangzhou 310020, China; schroyen@zuaa.zju.edu.cn

**Keywords:** gypenosides, STEC, ATF4-CHAC1-GPX4 axis, ferroptosis, gut microbiota

## Abstract

Shiga toxin-producing *Escherichia coli* (STEC) infection causes severe enteropathies and hemolytic uremic syndrome, yet non-antibiotic therapeutic strategies remain limited. This study evaluated whether gypenosides (GPs) protect against STEC-induced intestinal injury and investigated the underlying mechanisms. Mice were pretreated with GPs prior to STEC challenge, and protective effects were comprehensively assessed through survival analysis, bacterial burden quantification, transcriptomic profiling, flow cytometry, electron microscopy, and Western blotting. GPs significantly improved survival rates, alleviated diarrheal symptoms, reduced bacterial dissemination in systemic organs, and lowered Stx2 toxin levels. Furthermore, GPs effectively restored gut microbiota dysbiosis by enriching beneficial bacterial genera. Transcriptomic analysis suggested that GP treatment was associated with suppression of ferroptosis-related signaling pathways. Ultrastructural examination demonstrated ameliorated mitochondrial vacuolization, and biochemical assays confirmed decreased Fe^2+^ accumulation and attenuated lipid peroxidation. Mechanistically, these changes were associated with modulation of the ATF4-CHAC1-GPX4 axis and suppression of ferroptosis, together with preservation of mitochondrial integrity. Collectively, these findings identify GPs as a promising protective candidate against STEC-induced intestinal injury and highlight the ATF4-CHAC1-GPX4 axis as a candidate pathway for further mechanistic investigation.

## 1. Introduction

Shiga toxin-producing *Escherichia coli* (STEC) is a major foodborne pathogen causing severe diarrheal diseases, hemorrhagic colitis, and the life-threatening hemolytic uremic syndrome (HUS), with children under five years being particularly vulnerable to infection [1]. Conventional antibiotic therapy presents a significant clinical challenge because treatment induces bacterial SOS response, which enhances prophage-encoded Shiga toxin (Stx) expression and exacerbates toxin release, thereby increasing HUS incidence [2,3]. Compounding this therapeutic dilemma is the rising prevalence of antimicrobial resistance among pathogenic *E. coli* strains, which varies geographically and according to serotype [4,5]. While O157:H7 is the predominant serotype associated with human HUS, piglet-adapted STEC strains that carry virulence factors such as Stb, F18, AIDA, and Stx2e not only are economically important pathogens in veterinary medicine but also serve as valuable models for investigating Stx-mediated intestinal pathogenesis, as the downstream tissue injury pathways are shared across STEC pathotypes [6]. These converging clinical obstacles highlight the pressing need for alternative, non-antibiotic interventions.

Host gut microbiota composition critically determines susceptibility to STEC infection [7,8]. STEC colonization promotes expansion of the pathogenic *Escherichia–Shigella* genus while depleting commensal bacteria such as *Lactobacillus*, suggesting that microbiota restoration could serve as a promising adjunctive therapeutic approach [8,9]. Ferroptosis is iron-dependent cell death driven by lipid peroxidation, with glutathione peroxidase 4 (GPX4) serving as its central inhibitor [10]. Emerging evidence has identified ferroptosis as a key pathological mechanism in infectious enteropathies [11,12]. Specifically, enterotoxigenic *E. coli* induces ferroptosis through the downregulation of GPX4 and upregulation of CHAC1 [13]; whether STEC triggers this death pathway in vivo remains unknown. Activating transcription factor 4 (ATF4) transcriptionally activates CHAC1, leading to glutathione depletion and subsequent ferroptotic cell death [14]. ATF4 additionally modulates mitophagy, and excessive mitophagic activity has been implicated in intestinal barrier disruption [15,16]. Nevertheless, the potential crosstalk between ferroptosis and mitophagy in STEC-induced enteropathy, and whether targeting the ATF4-CHAC1-GPX4 axis can simultaneously inhibit both pathways, has not been investigated.

Natural saponins derived from medicinal plants have garnered increasing attention as safe and multi-target bioactive agents [17]. Gypenosides (GPs), the major bioactive saponins from the traditional herb *Gynostemma pentaphyllum*, possess antioxidant, anti-inflammatory, and gut-protective properties [18,19]. Previous investigations demonstrated that GPs improve intestinal barrier function and ameliorate dextran sulfate sodium (DSS)-induced colitis in murine models [20], yet their efficacy against STEC infection has not been evaluated.

In this study, we demonstrate that GPs protect against STEC infection in mice by improving survival, alleviating diarrhea, reducing bacterial colonization, and restoring gut microbiota dysbiosis. Mechanistic investigations revealed that GP treatment was associated with modulation of the ATF4-CHAC1-GPX4 axis, alongside suppression of ferroptosis and preservation of mitochondrial integrity, and attenuation of oxidative stress. These findings identify the ATF4-CHAC1-GPX4 axis as a candidate pathway warranting further mechanistic validation and position GPs as a promising non-antibiotic protective agent for STEC management.

## 2. Materials and Methods

### 2.1. Chemicals and Reagents

Gypenosides (GPs) with a purity of ≥95% were purchased from Shanghai Yuanye Bio-Technology Co., Ltd. (Shanghai, China; Lot No. H23a9k59541). Cell culture reagents including Luria–Bertani (LB) medium, DMEM, fetal bovine serum (FBS), penicillin–streptomycin, lipopolysaccharide (LPS) and donkey anti-rabbit IgG (H+L) antibody were obtained from Thermo Fisher Scientific Inc. (Shanghai, China). The cell counting kit-8 (CCK-8) assay kit was purchased from Dojindo Laboratories (Shanghai, China). ELISA kits for superoxide dismutase (SOD), catalase (CAT), malondialdehyde (MDA), cyclooxygenase-2 (COX-2), total antioxidant capacity (T-AOC), glutathione (GSH), glutathione peroxidase (GSH-Px), glutathione disulfide (GSSG), diamine oxidase (DAO) and D-lactic acid (DLA) were obtained from Jiangsu Meimian Industrial Co., Ltd. (Nanjing, China). The Fe^2+^ assay kit and DAPI solution were purchased from Solarbio Science & Technology Co., Ltd. (Beijing, China). RNA extraction and PCR reagents including the RNAsimple Total RNA Extraction, PrimeScript™ RT Master Mix, and TB Green^®^ Premix Ex Taq™ II kits were obtained from Tiangen and Takara (Beijing, China). Protein extraction and detection reagents including RIPA lysis buffer, the BCA Protein Assay Kit, and an enhanced chemiluminescence (ECL) reagent kit were purchased from Beyotime and Sangon Biotech (Shanghai, China). Primary antibodies were obtained from the following sources: ZO-1, ATF4, CHAC1, GPX4, and GAPDH from Proteintech (Wuhan, China); Claudin-1 from Abcam (Shanghai, China); and FITC-conjugated anti-CD3e (145-2C11), PE-Cy7-conjugated anti-CD4 (GK1.5), APC-Cy7-conjugated anti-CD8a (53-6.7), APC-conjugated anti-CD19 (1D3), PE-conjugated anti-NK1.1 (PK136), and APC-conjugated anti-CD11c (N418) from MultiSciences (Hangzhou, China). Dihydroethidium (DHE) staining solution was from Sigma-Aldrich (Shanghai, China).

### 2.2. Bacterial and Cell Culture

The Shiga toxin-producing *Escherichia coli* (STEC) strain used in this study was isolated from the intestinal contents of diarrheic piglets and is currently maintained in our laboratory. This STEC strain harbors the following virulence factors: Stb, F18, AIDA and Stx2e. Prior to experimentation, the STEC strain was cultured on LB agar plates. A single colony was picked and inoculated into LB broth, then incubated at 37 °C with shaking to obtain a bacterial suspension in the log phase.

MODE-K is a murine small intestinal epithelial cell line immortalized by expression of the SV40 large T antigen, and its data are available in the Cellosaurus database under accession number CVCL_B4FG. The cell line was maintained in our laboratory and cultured in Dulbecco’s Modified Eagle’s Medium (DMEM) supplemented with 10% FBS and 1% penicillin–streptomycin at 37 °C in a 5% CO_2_ atmosphere.

### 2.3. Cell Viability Assay

MODE-K cells were seeded in 96-well culture plates at a density of 1 × 10^5^ cells/mL and cultured overnight. For dose–response analysis, 100 μL of DMEM containing different concentrations of GPs (1600, 1400, 1200, 1000, 800, 600, 400, 200, 100, 50, and 25 μg/mL) was added, with control and blank wells included. Cells were incubated for 24 h, and viability was assessed using the CCK-8 assay kit according to the manufacturer’s instructions as previously described [21]. For the protective effect evaluation, MODE-K cells were seeded in 96-well plates and cultured overnight. Cells were pretreated with 100 μL of medium containing 600 μg/mL GPs for 24 h. STEC cultures in the logarithmic growth phase were then prepared and used to infect the cells at a multiplicity of infection (MOI) of 100 for 2 h. After washing with PBS, cell viability was assessed using the CCK-8 assay kit.

### 2.4. Animal Experiments

Healthy male specific-pathogen-free (SPF)-grade BALB/c mice weighing approximately 10–12 g were purchased from Shanghai SLAC Laboratory Animal Co., Ltd. (Shanghai, China) and maintained under controlled conditions. All animal procedures were approved by the Animal Care Committee of the Zhejiang Academy of Agricultural Sciences (approved protocol #: ZAASLA2025092402; approved date: 24 September, 2025).

For the survival study, mice were randomly divided into five groups (*n* = 20 per group): the control group (saline), the STEC-infected group (STEC), and three GP-treated groups (STEC + 400 mg/kg GPs, STEC + 200 mg/kg GPs, and STEC + 100 mg/kg GPs). From day 1 to day 7, the saline and STEC groups received daily oral gavage with 0.5 mL physiological saline, while the three GP-treated groups were administered 0.5 mL of GP suspension at doses of 400, 200, and 100 mg/kg, respectively. Two hours after the final gavage on day 7, mice in the STEC and three GP-treated groups were intraperitoneally injected with 0.5 mL of STEC bacterial suspension (1 × 10^9^ CFU/mL), whereas the saline group received 0.5 mL physiological saline. After STEC infection, diarrheal symptoms were monitored daily, and the survival of mice was recorded for 7 consecutive days to generate survival curves.

For sample collection, mice were randomly divided into three groups (*n* = 15 per group): saline, STEC, and STEC + GP (100 mg/kg) groups. The treatment protocol was identical to the survival study except that mice received 0.2 mL of STEC suspension (1 × 10^9^ CFU/mL) or saline at day 7. This lower dose was selected based on preliminary experiments to ensure a sufficient number of surviving animals for downstream analyses. At 24 h post-infection, 5 surviving mice were randomly selected from each group and euthanized for sample collection. Blood was collected for serum separation (stored at −80 °C). Liver, spleen, and fecal samples were obtained for bacterial load determination. Intestinal tissues (duodenum, jejunum, ileum, and colon) were harvested for histopathological and ultrastructural analysis. Additionally, spleen, mesenteric lymph nodes (MLNs), and intestinal tissues were snap-frozen in liquid nitrogen and stored at −80 °C for further molecular analysis.

### 2.5. Bacterial Load Determination

Bacterial load was determined by the standard plate count method as previously described [22]. Liver, spleen, and fecal samples were aseptically collected and homogenized in PBS at a 1:10 (*w*/*v*) ratio using sterile tissue grinders. The homogenates were serially diluted 10-fold in PBS, plated onto MacConkey agar plates, and incubated at 37 °C for 16 h. Bacterial colonies were enumerated and expressed as colony-forming units per gram of tissue or feces (CFU/g).

### 2.6. Blood Analysis

Collected blood samples were transferred to EDTA-anticoagulant tubes and analyzed using an automated hematology analyzer to determine total white blood cell count (WBC) and differential leukocyte counts, including percentages of neutrophils, lymphocytes, monocytes, and eosinophils.

### 2.7. Gut Microbiota Analysis

Fecal samples were collected from mice of different groups and snap-frozen in liquid nitrogen. Genomic DNA was extracted from the fecal samples. After amplification and quality control, PCR products were pooled, purified, and used for library construction. Sequencing was performed by Novogene Co., Ltd. (Beijing, China) on an Illumina platform for paired-end sequencing.

### 2.8. Flow Cytometry Analysis

Flow cytometric analysis of lymphocyte populations was performed according to established protocols [23]. Mononuclear cells were isolated from the spleen and MLNs and subjected to surface marker staining. The abovementioned antibodies were used for surface marker staining. The analysis was performed by a FACS Canto™ (BD Biosciences, San Diego, CA, USA) and FlowJo (v10.0).

### 2.9. Histological Analysis

Histological examination was conducted following standard H&E staining protocol [24,25]. The duodenum, jejunum, ileum, and colon were fixed in 4% paraformaldehyde for 24 h. After washing, the samples were processed through a graded ethanol series (75%, 85%, 95%, and absolute ethanol), cleared in xylene, and embedded in paraffin. Sections were deparaffinized, rehydrated, stained with hematoxylin for 4 min, differentiated in 0.8% acid–alcohol for 2 s, counterstained with eosin for 20 s, dehydrated, cleared, and mounted. Tissue morphology was examined under a microscope (Olympus Corporation, Tokyo, Japan), and digital images were captured using an imaging system (Hamamatsu Photonics K.K., Hamamatsu City, Japan).

### 2.10. Electron Microscopy

For ultrastructural analysis, intestinal tissues were fixed in 2.5% glutaraldehyde overnight, post-fixed in 1% osmium tetroxide for 2 h, and dehydrated through a graded ethanol series. For scanning electron microscopy (SEM), samples were then immersed in isoamyl acetate for 15 min, dried, sputter-coated with gold for 30 s, and examined using a Hitachi TM3000 scanning electron microscope (Hitachi High-Tech Corporation, Tokyo, Japan). For transmission electron microscopy (TEM), samples were embedded in epoxy resin. Ultrathin sections (70 nm) were cut, stained with uranyl acetate and lead citrate, and examined under a Hitachi HT7800 transmission electron microscope (Hitachi High-Tech Corporation, Tokyo, Japan).

### 2.11. Immunofluorescence and DHE Staining

Immunofluorescence staining was performed following manufacturer protocols [26]. Paraffin-embedded sections were deparaffinized and rehydrated, and underwent antigen retrieval by high-pressure heating in EDTA buffer (pH 9.0). After blocking with 10% donkey serum for 30 min at 37 °C, sections were incubated overnight at 4 °C with primary antibodies against ZO-1 (1:1500) and Claudin-1 (1:1500). Following washing, sections were incubated with secondary antibody at 37 °C for 45 min. Nuclei were counterstained with DAPI for 5 min in the dark. Slides were mounted with anti-fade mounting medium and imaged as described above.

For DHE staining, snap-frozen duodenum tissues were sectioned using a cryostat. Sections were stained with DAPI for 2 min, washed, and incubated with DHE staining solution at room temperature in the dark for 1 h. After washing, slides were mounted and observed as described above.

### 2.12. Biochemical Assays

The levels of SOD, CAT, MDA, COX-2, T-AOC, GSH, GSH-Px, GSSG, DAO, DLA, and LPS in mouse serum and duodenum were measured using ELISA kits according to the manufacturers’ instructions. The ferrous ion (Fe^2+^) content in mouse duodenum was measured using a commercial Fe^2+^ assay kit according to the manufacturer’s instructions.

### 2.13. Transcriptome Analysis

MLN samples from all experimental groups were collected and snap-frozen in liquid nitrogen. Total RNA was extracted and quality was assessed with the RNA Nano 6000 Assay Kit from the Bioanalyzer 2100 system (Agilent Technologies, Santa Clara, CA, USA). Library preparation and transcriptome sequencing were performed by Novogene Co., Ltd. (Beijing, China). Differentially expressed genes (DEGs) were identified using a threshold of |log_2_(fold change)| > 1 and adjusted *p*-value < 0.05. KEGG pathway enrichment analyses were conducted using the clusterProfiler R package (version 3.8.1). Raw sequencing data generated in this study have been deposited in the NCBI Sequence Read Archive (SRA) under BioProject accession number PRJNA1499905 (https://www.ncbi.nlm.nih.gov/sra/PRJNA1499905, accessed on 24 July 2026).

### 2.14. Quantitative Real-Time PCR (qPCR)

Total RNA was extracted from tissues and MODE-K cells. RNA was reverse-transcribed into cDNA and the relative mRNA expression of IL-10, IL-1β, IL-6, IL-18, TNF-α, Stx2, GPX4, PTGS2, ATF4, CHAC1 and ACSL4 was detected by qPCR, with GAPDH as the internal reference gene. Primer sequences are listed in Supplementary Table S1. The relative mRNA expression levels were calculated using the 2^−ΔΔCT^ method.

### 2.15. Western Blot Analysis

Proteins were extracted from duodenum tissues using RIPA lysis buffer. Protein concentration was quantified using a BCA Protein Assay Kit, and equal amounts of protein extracts were separated by SDS-PAGE and transferred onto PVDF membranes. Membranes were blocked with 5% skim milk and incubated overnight at 4 °C with primary antibodies against ATF4, CHAC1, GPX4, and GAPDH. After washing, membranes were incubated with secondary antibody for 90 min at room temperature. Protein bands were visualized using an ECL reagent kit and captured by a gel imaging system (Bio-Rad Laboratories, Inc., Hercules, CA, USA). Due to overlapping molecular weights of GAPDH and the target proteins, separate gels were run simultaneously under identical conditions, and all membranes were exposed for the same duration. Densitometric analysis was performed using ImageJ software (version 1.53t), and values were normalized to GAPDH. Western blot analysis was performed with three independent biological replicates.

### 2.16. Statistical Analysis

All data were expressed as mean ± standard deviation (SD). Statistical analyses were performed with GraphPad Prism software (version 9.5.0, GraphPad Software, Inc., San Diego, CA, USA). The following statistical methods were applied. Survival curves were analyzed using the Kaplan–Meier method with the log-rank (Mantel–Cox) test. For gut microbiota, α-diversity was compared by the Wilcoxon rank-sum test; β-diversity was assessed by principal component analysis (PCA) based on Bray–Curtis distances, with group differences tested by PERMANOVA (Adonis); differential abundance at the phylum and genus levels was analyzed using the Kruskal–Wallis test with false discovery rate (FDR) correction. For transcriptomic data, differential expression analysis was performed using DESeq2 with an adjusted *p*-value < 0.05 and |log_2_(fold change)| > 1; KEGG pathway enrichment analysis was performed using the hypergeometric test with FDR correction, and pathways with *p* < 0.05 were considered significantly enriched. Other continuous variables were analyzed using one-way ANOVA with Tukey’s multiple comparisons test; *p* < 0.05 was considered statistically significant.

## 3. Results

### 3.1. GPs Alleviated STEC-Induced Pathogenicity

**Figure 1:**
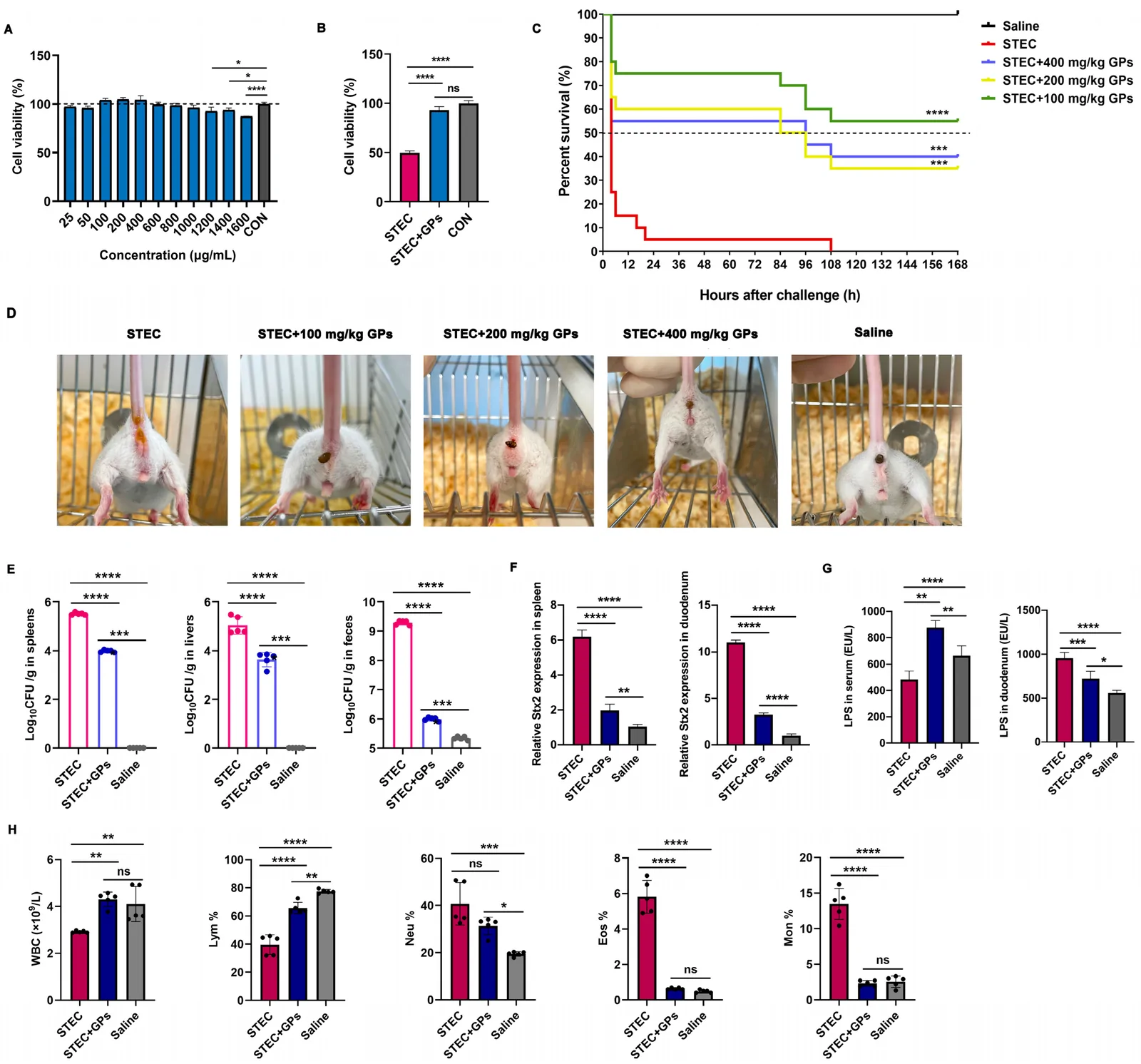
GPs alleviated STEC-induced pathogenicity. (**A**) Cell viability of MODE-K cells treated with GPs at various concentrations for 24 h. (**B**) Effects of GP pretreatment on STEC-induced decrease in MODE-K cell viability. (**C**) Survival curves of mice infected with STEC and treated with GPs at different doses (*n* = 20). *** *p* < 0.001, **** *p* < 0.0001 vs. STEC group. (**D**) Representative images of fecal consistency in STEC-infected mice treated with GPs. (**E**) Bacterial colonization of STEC in spleen, liver, and feces of mice. (**F**) Shiga toxin Stx2 levels in spleen and duodenum of STEC-infected mice treated with GPs. (**G**) LPS levels in serum and duodenum of STEC-infected mice treated with GPs. (**H**) Hematological parameters including total white blood cell (WBC) count and differential leukocyte percentages in STEC-infected mice after GP pretreatment. For panels (**E**–**H**), data are presented as mean ± SD (*n* = 5). * *p* < 0.05, ** *p* < 0.01, *** *p* < 0.001, **** *p* < 0.0001. ns, no significant difference.

As shown in Figure 1A, treatment of MODE-K cells with GPs at concentrations ranging from 25 to 1600 μg/mL for 24 h did not significantly alter cell viability. Notably, cell viability was slightly increased at concentrations of 1200–1600 μg/mL, indicating that GPs exerted no cytotoxic effects within the tested concentration range. STEC infection significantly reduced cell viability compared with the saline group; however, pretreatment with 600 μg/mL GPs for 24 h significantly attenuated the STEC-induced decrease in cell viability (Figure 1B).

As demonstrated in Figure 1C, STEC infection was highly virulent, reducing mouse survival rates to 5% within 24 h post-infection, with 100% mortality observed by 108 h. In contrast, GP pretreatment significantly improved survival rates of infected mice. Specifically, survival rates in the STEC + 400 mg/kg GPs group were 55% at 24 h and declined to 40% by 168 h. Similarly, the STEC + 200 mg/kg GPs group exhibited survival rates of 60% and 35% at 24 h and 168 h, respectively. Notably, the STEC + 100 mg/kg GPs group exhibited the highest survival rates, maintaining 75% at 24 h and maintaining 55% at 168 h. As shown in Figure 1D, STEC-infected mice exhibited typical diarrheal symptoms, characterized by loose and watery stools, whereas GP pretreatment at doses of 100, 200, and 400 mg/kg significantly alleviated diarrhea and restored fecal consistency. Consistently, GP pretreatment significantly reduced bacterial colonization in the spleen, liver, and feces, indicating that GPs effectively reduced STEC dissemination and proliferation (Figure 1E). Similarly, GP pretreatment significantly reduced Stx2 levels in both the spleen and duodenum (Figure 1F). However, divergent effects were observed for LPS content across different tissues: GP pretreatment significantly decreased LPS levels in the duodenum but elevated LPS levels in the serum (Figure 1G). While the reduction in duodenal LPS reflects improved local barrier function, the elevated serum LPS may indicate redistribution of this endotoxin from the intestinal pool into the circulation, without producing overt pathology. Furthermore, compared with the saline group, mice in the STEC group showed significantly reduced total white blood cell (WBC) count and lymphocyte percentage, together with increased proportions of neutrophils, monocytes, and eosinophils. GP pretreatment significantly increased both total WBC count and lymphocyte percentage while reducing the proportions of neutrophils, monocytes, and eosinophils compared with the STEC group; however, the reduction in neutrophils did not reach statistical significance (Figure 1H).

### 3.2. GPs Alleviated STEC-Induced Gut Microbiota Dysbiosis

**Figure 2:**
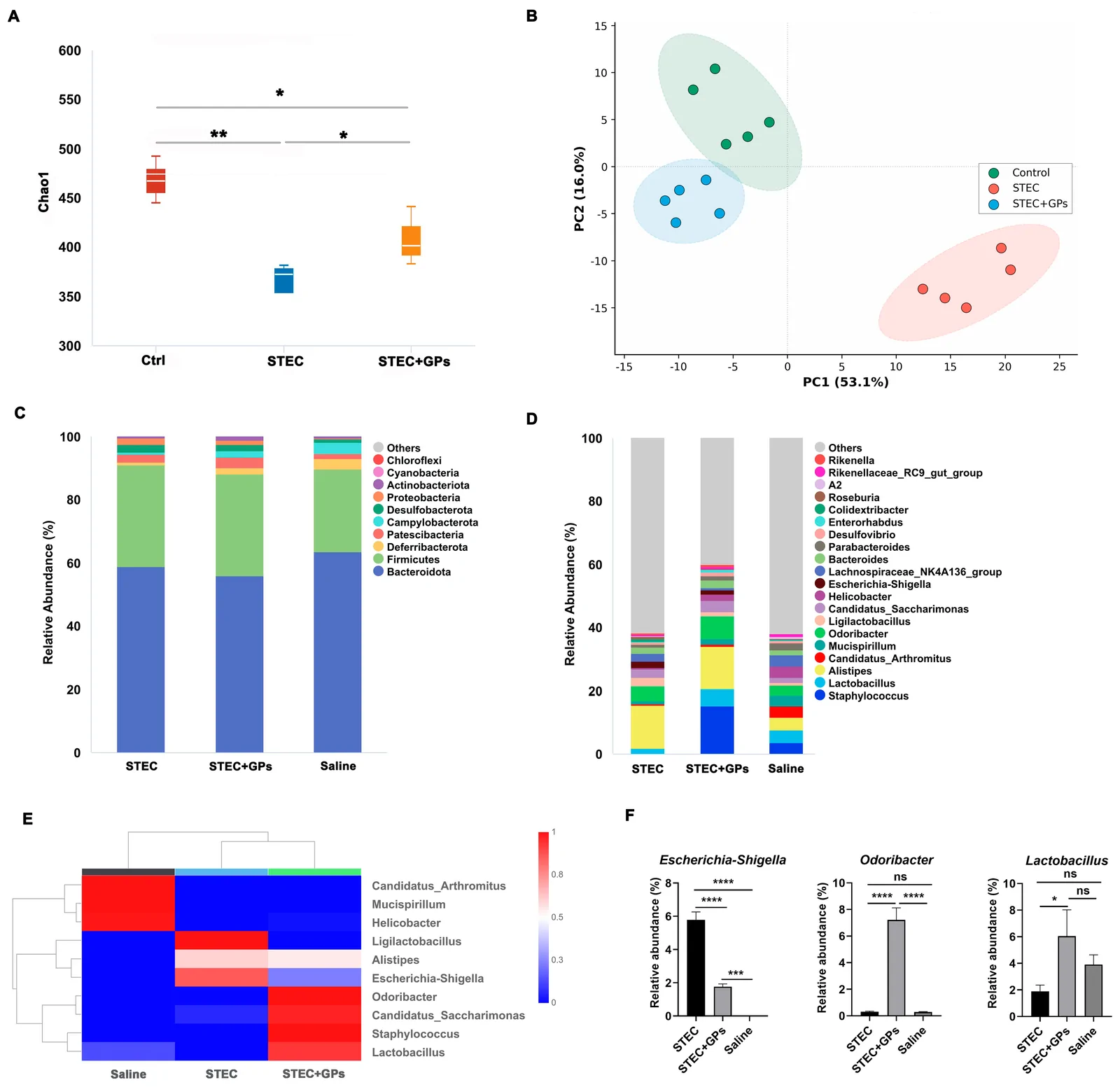
Effects of GPs on gut microbiota dysbiosis induced by STEC infection in mice. (**A**) α-diversity of gut microbiota assessed by the Chao1 index (*n* = 5). (**B**) β-diversity of gut microbiota visualized by principal component analysis (PCA). (**C**) Relative abundance of gut microbiota at the phylum level. (**D**) Relative abundance of gut microbiota at the genus level. (**E**) Heatmap showing the relative abundance of gut microbiota at the genus level. (**F**) Relative abundance of *Escherichia-Shigella*, *Odoribacter* and *Lactobacillus*. * *p* < 0.05, ** *p* < 0.01, *** *p* < 0.001, **** *p* < 0.0001. ns, no significant difference.

Analysis of α-diversity in mouse gut microbiota revealed that the Chao1 index was significantly lower in the STEC-infected group compared to the saline group at 24 h post-infection, indicating that STEC infection reduced species richness of the gut microbiota. Notably, GP pretreatment significantly attenuated this reduction, suggesting its potential to restore gut microbial diversity (Figure 2A). β-diversity analysis showed clear separation between the STEC-infected, saline, and GP-pretreated groups in PCA space, with the GP-pretreated group clustering more closely to the saline group. These results indicate that STEC infection substantially alters the overall structure of the gut microbiota, and GP pretreatment effectively mitigates these alterations (Figure 2B). At the phylum level, *Firmicutes* and *Bacteroidetes* were the dominant bacterial phyla across all groups (Figure 2C). At the genus level, the relative abundance of the *Escherichia-Shigella* genus was significantly increased in the STEC-infected group (Figure 2D). However, following GP pretreatment, the relative abundance of *Escherichia-Shigella* was markedly decreased, while that of beneficial bacteria such as *Lactobacillus* and *Odoribacter* was significantly increased (Figure 2E,F).

### 3.3. GPs Alleviated STEC-Induced Abnormal Immune Cell Responses

**Figure 3:**
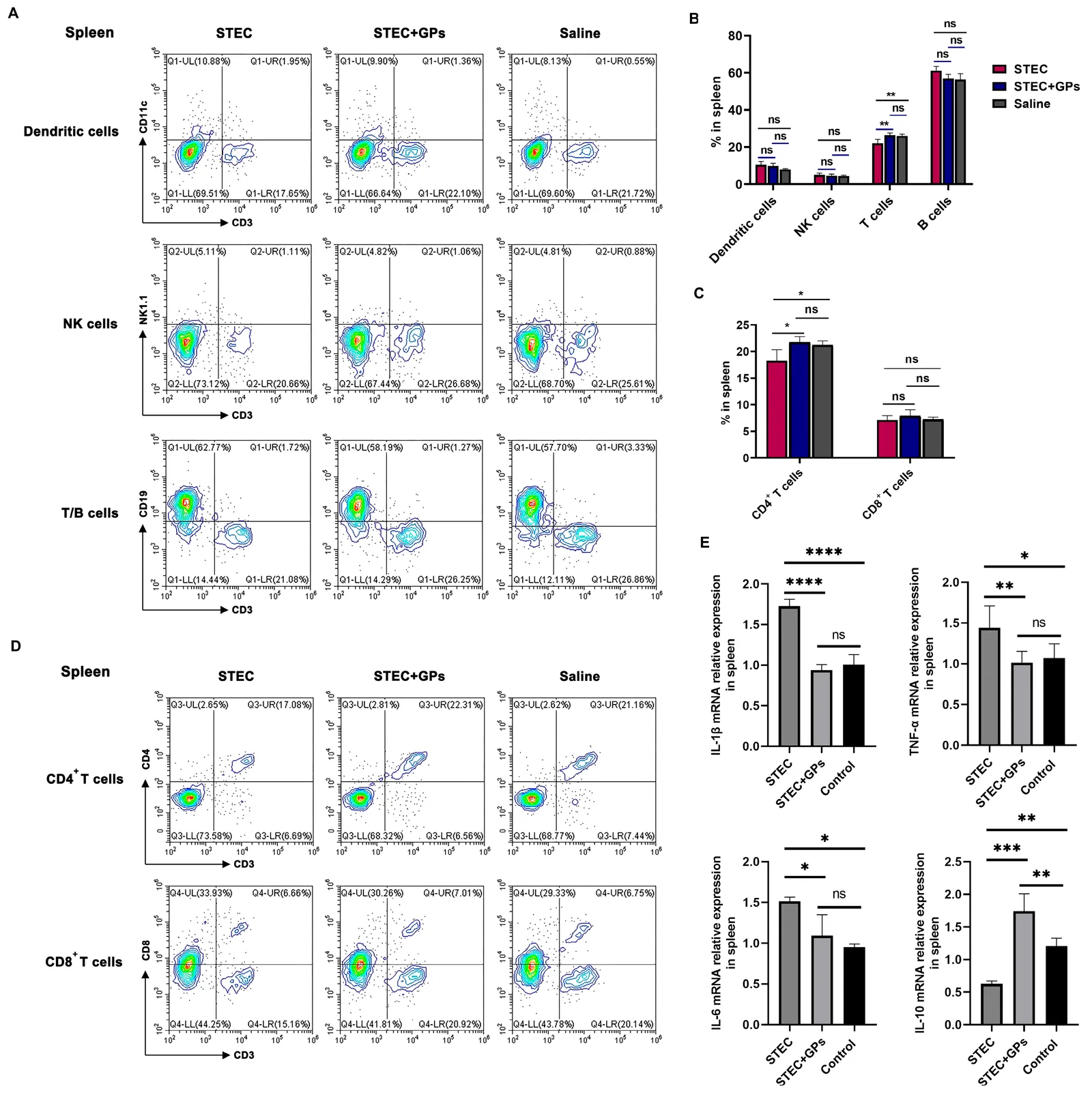
Effects of GP pretreatment on STEC-induced immune cell alterations and cytokine gene expression in the spleen. (**A**) Flow cytometric analysis of splenic immune cell populations (*n* = 5). (**B**) Proportions of dendritic cells, NK cells, T cells and B cells in the spleen. (**C**) Proportions of CD4^+^ T cells and CD8^+^ T cells. (**D**) Subset analysis of splenic T cells. (**E**) mRNA expression levels of cytokines in the spleen. * *p* < 0.05, ** *p* < 0.01, *** *p* < 0.001,**** *p* < 0.0001. ns, no significant difference.

Flow cytometric analysis of splenic tissues showed that, compared to the saline control group, STEC infection caused a significant decrease in the proportion of splenic T cells, while no obvious effects were observed on the proportions of dendritic cells, NK cells, or B cells (Figure 3A,B). Pretreatment with GPs significantly reversed the STEC-induced reduction in splenic T cell proportion, resulting in no statistically significant difference compared with the saline control group. Further subset analysis demonstrated that, relative to the saline control group, STEC infection primarily led to a significant decrease in the proportion of splenic CD4^+^ T cell subsets, with no obvious effect on CD8^+^ T cell subsets (Figure 3C,D). GP pretreatment significantly ameliorated the STEC-induced reduction in CD4^+^ T cell subset proportion, restoring it to a level comparable with that of the saline control group, but exerted no obvious regulatory effect on CD8^+^ T cell subsets. Cytokine expression analysis revealed that, compared with the saline control group, STEC infection significantly upregulated the mRNA expression levels of pro-inflammatory cytokines IL-1β, TNF-α, and IL-6 in the spleen, while simultaneously suppressing the gene expression of the anti-inflammatory cytokine IL-10 (Figure 3E). GP pretreatment effectively alleviated this STEC-induced cytokine imbalance by significantly downregulating the mRNA expression of IL-1β, TNF-α, and IL-6, and upregulating IL-10 expression, such that all aforementioned parameters showed no significant difference compared with the saline control group.

**Figure 4:**
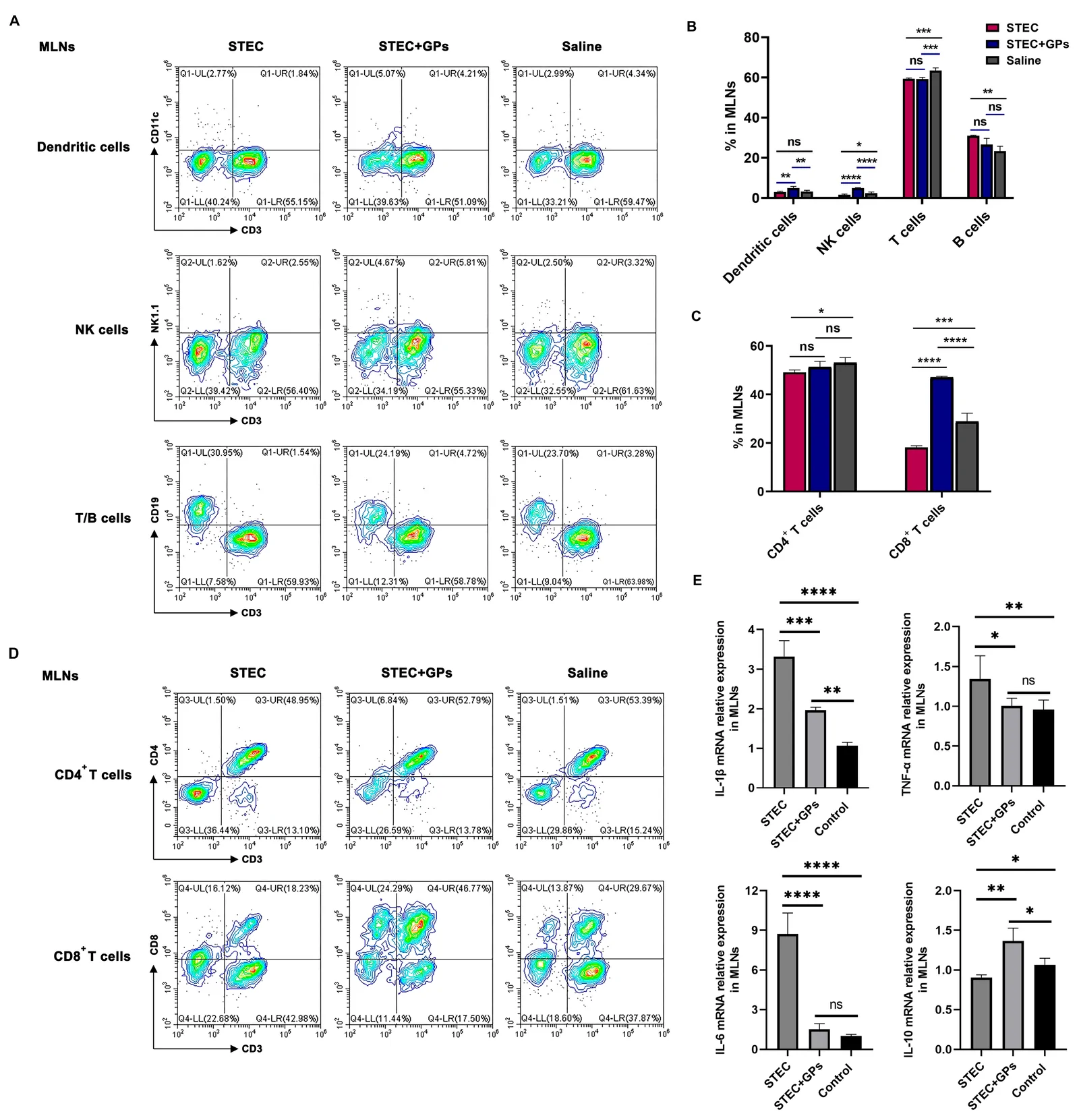
Effects of GP pretreatment on STEC-induced immune cell alterations and cytokine gene expression in MLNs. (**A**) Flow cytometric analysis of immune cell populations in MLNs (*n* = 5). (**B**) Proportions of dendritic cells, NK cells, T cells and B cells in MLNs. (**C**) Proportions of CD4^+^ T cells and CD8^+^ T cells. (**D**) Subset analysis of T cells in MLNs. (**E**) mRNA expression levels of cytokines in MLNs. * *p* < 0.05, ** *p* < 0.01, *** *p* < 0.001,**** *p* < 0.0001. ns, no significant difference.

Flow cytometric analysis of MLNs indicated that, compared with the saline control group, STEC infection caused significant decreases in the proportions of NK cells and T cells, a marked increase in B cell proportion, and no obvious effect on the proportion of dendritic cells (Figure 4A,B). GP pretreatment significantly reversed the STEC-induced reduction in NK cell proportion in MLNs; notably, the NK cell proportion in the GP pretreatment group was significantly higher than that in the saline control group. Furthermore, GP pretreatment also significantly increased the proportion of dendritic cells in MLNs, which was likewise significantly higher than that in the saline control group. However, GP pretreatment exerted no obvious ameliorative effect on the STEC-induced changes in T cell and B cell proportions in MLNs. Further subset analysis demonstrated that, compared with the saline control group, STEC infection caused significant reductions in both CD4^+^ and CD8^+^ T cell subset proportions in MLNs (Figure 4C,D). GP pretreatment significantly alleviated the STEC-induced reduction in CD8^+^ T cell subset proportion, resulting in a level significantly higher than that of the saline control group, but had no obvious regulatory effect on CD4^+^ T cell subsets. Regarding cytokine expression patterns, consistent with the findings in splenic tissues, STEC infection significantly upregulated the mRNA expression of IL-1β, TNF-α, and IL-6 in MLNs, while simultaneously markedly inhibiting IL-10 expression (Figure 4E). GP pretreatment effectively alleviated this cytokine imbalance in MLNs by significantly suppressing the mRNA expression levels of IL-1β, TNF-α, and IL-6, and upregulating IL-10 mRNA expression. Notably, the gene expression levels of TNF-α and IL-6 in the GP pretreatment group showed no significant difference compared with the saline control group, whereas the gene expression levels of IL-1β and IL-10 were both significantly higher than those in the saline control group.

### 3.4. GPs Alleviated Pathological Damage in the Intestines of STEC-Infected Mice

**Figure 5:**
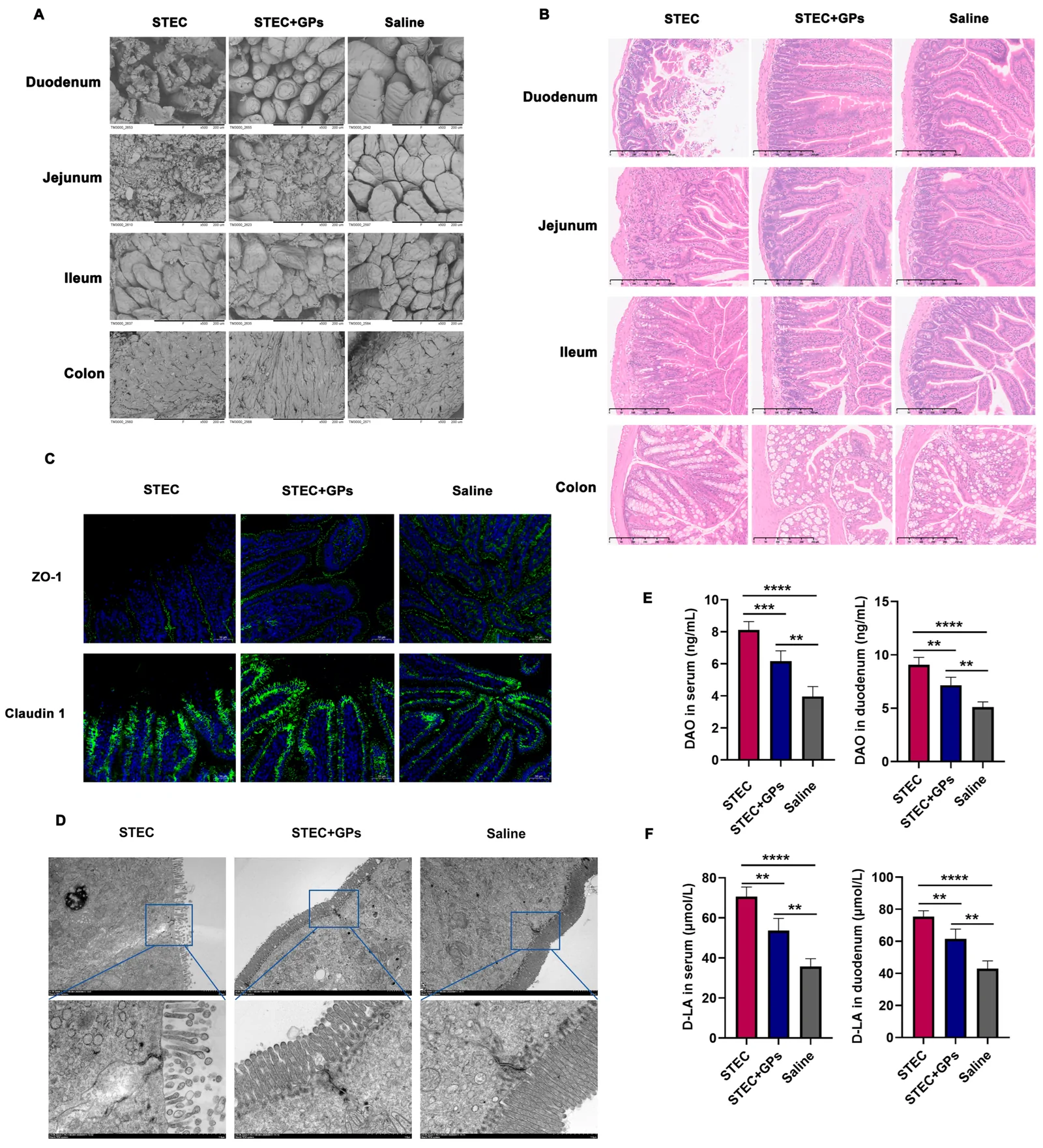
Protective effects of GPs against intestinal barrier injury in STEC-infected mice. (**A**) Ultrastructural changes in intestinal villi observed by SEM. Scale bar = 200 μm. (**B**) Histopathological alterations in intestinal tissues evaluated by HE staining. Scale bar = 250 μm. (**C**) Expression of tight junction proteins ZO-1 and Claudin-1 (green). Scale bar = 50 μm. (**D**) Ultrastructure of intestinal tight junctions. Scale bar = 2 μm. (**E**) DAO levels in serum and duodenal tissues. (**F**) D-LA levels in serum and duodenal tissues. ** *p* < 0.01, *** *p* < 0.001,**** *p* < 0.0001.

Ultrastructural changes in the intestinal villi were examined by SEM. As demonstrated in Figure 5A, compared with the saline group, the STEC-infected group displayed severe structural damage in the intestinal villi, particularly in the duodenum and jejunum, characterized by rupture and shedding of villi. Notably, GP pretreatment significantly restored the structural integrity of the villi in both the duodenum and jejunum, markedly alleviating the extent of tissue damage. Histopathological changes in the intestinal tissues of mice induced by the STEC strain were evaluated via HE staining to assess the protective effect of GPs. As shown in Figure 5B, consistent with the SEM observations, STEC-infected mice exhibited significant pathological injuries in the duodenum and jejunum, including necrosis and exfoliation of the duodenal mucosal epithelium, thickening of the lamina propria, exposed villi, and hyperplasia of inflammatory cells, as well as focal necrosis, loss of small intestinal glands, and disorganized tissue architecture in the jejunum. In contrast, GP pretreatment markedly ameliorated these histopathological alterations in both the duodenum and jejunum, presenting intact villi with orderly arrangement, uniform mucosal thickness, well-preserved epithelial morphology, and absence of significant inflammatory infiltration. Notably, no obvious pathological changes were observed in the ileum or colon across all groups. To further evaluate the integrity of the intestinal barrier, the expression of tight junction proteins ZO-1 and Claudin-1 in duodenal tissues was examined by immunofluorescence staining. As illustrated in Figure 5C, ZO-1 and Claudin-1 proteins (green) exhibited continuous and intact distributions in the saline group. In contrast, STEC infection markedly reduced the expression of both proteins, resulted in discontinuous distribution, and significantly weakened fluorescence intensity, indicating severe disruption of the tight junction structure. Compared with the STEC group, the GP pretreatment group demonstrated significantly increased expression abundance of ZO-1 and Claudin-1, improved distribution continuity, and markedly enhanced fluorescence intensity. These results are consistent with the ultrastructural observations of intestinal tight junctions (Figure 5D), suggesting that STEC infection disrupts intestinal tight junction structure, whereas GP pretreatment significantly mitigates this disruptive effect. DAO and D-LA are well-established biomarkers reflecting intestinal barrier function integrity. DAO is a specific enzyme localized to the intestinal mucosal brush border, primarily reflecting the degree of intestinal mucosal damage; D-LA is a bacterial metabolite that enters the circulation when the intestinal barrier is compromised and permeability is increased. As shown in Figure 5E,F, STEC infection led to significantly elevated levels of both DAO and D-LA in blood and duodenal tissues, indicating intestinal barrier injury. In contrast, GP pretreatment substantially attenuated this elevation, further confirming the protective effect of GPs on intestinal barrier function.

### 3.5. GPs Alleviated Oxidative Stress and Mitochondrial Damage Induced by STEC Infection in Mice

**Figure 6:**
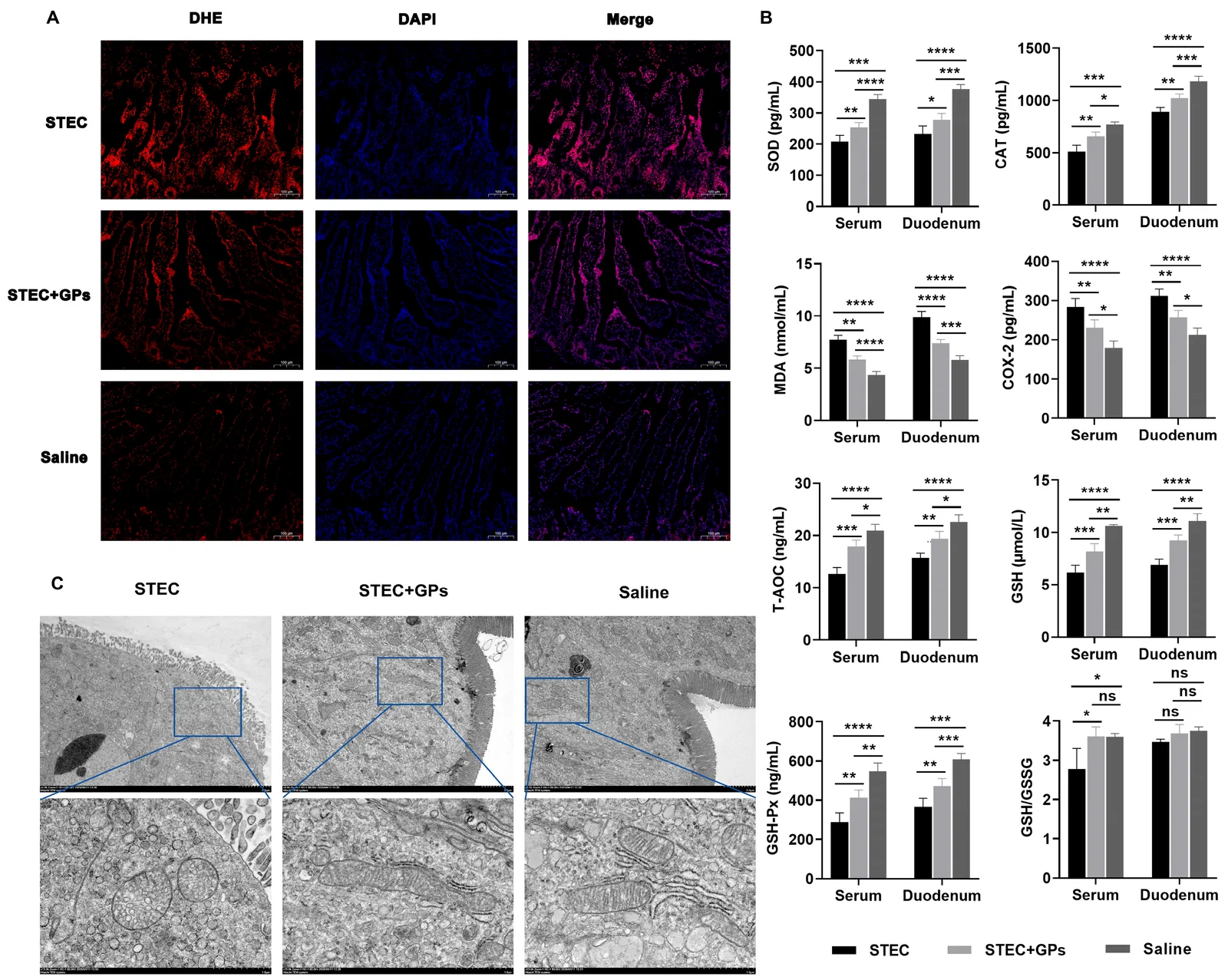
GPs alleviate oxidative stress and mitochondrial damage in the duodenum of STEC-infected mice. (**A**) ROS levels in duodenal tissues detected by DHE immunofluorescence staining (red). Scale bar = 100 μm. (**B**) Levels of oxidative stress-related indicators in serum and the duodenum. (**C**) Ultrastructure of duodenal mitochondria observed by SEM. Scale bar = 2 μm. * *p* < 0.05, ** *p* < 0.01, *** *p* < 0.001, **** *p* < 0.0001. ns, no significant difference.

Reactive oxygen species (ROS) are highly reactive oxygen metabolites that can cause oxidative damage to cellular macromolecules when accumulated beyond the capacity of antioxidant defenses. DHE is a fluorescent probe that reacts with intracellular superoxide anions to generate red fluorescence, allowing ROS levels to be quantified. DHE immunofluorescence staining of the duodenum revealed a pronounced increase in fluorescence intensity in the STEC group, indicating substantial ROS accumulation induced by STEC infection (Figure 6A). In contrast, GP treatment markedly attenuated the DHE signal, demonstrating its efficacy in alleviating ROS generation. SOD, CAT, and GSH-Px are classic antioxidant enzymes whose activities indirectly reflect the organism’s capacity to resist free radical damage. MDA, the terminal product of lipid peroxidation induced by free radicals, reflects the extent of lipid peroxidation damage. T-AOC represents the overall antioxidant capacity of the organism. GSH is a key component of the non-enzymatic antioxidant system, and the GSH/GSSG ratio serves as a critical indicator for evaluating cellular oxidative stress status. COX-2 is an important marker of inflammatory and oxidative stress responses. As shown in Figure 6B, compared with the saline group, STEC-infected mice exhibited significantly elevated levels of MDA and COX-2 in both serum and duodenal tissues. Concurrently, the levels of SOD, CAT, T-AOC, GSH, and GSH-Px were significantly decreased. Furthermore, the serum GSH/GSSG ratio was significantly reduced, indicating that STEC infection induces oxidative stress. In contrast, the GP treatment group showed a significant reduction in MDA and COX-2 levels in both serum and duodenal tissues compared with the STEC group, while the levels of SOD, CAT, T-AOC, GSH, and GSH-Px were significantly restored. Additionally, the serum GSH/GSSG ratio was significantly increased, suggesting that GPs effectively alleviate oxidative stress damage induced by STEC infection.

Ultrastructural examination of the duodenum by SEM revealed that STEC infection induced marked mitochondrial swelling with cristae disruption, vacuolization, and extensive immune cell infiltration, exhibiting clear signs of mitochondrial damage (Figure 6C). In contrast, GP pretreatment markedly mitigated STEC-induced mitochondrial injury, with significant improvement in mitochondrial structural integrity. As mitochondria are the primary source of intracellular ROS, their damage can further exacerbate oxidative stress. The protective effect of GPs may be related to preservation of mitochondrial function and reduction of ROS production.

### 3.6. GPs Alleviated STEC Infection-Induced Intestinal Damage in Association with Suppression of the Ferroptosis Pathway

**Figure 7:**
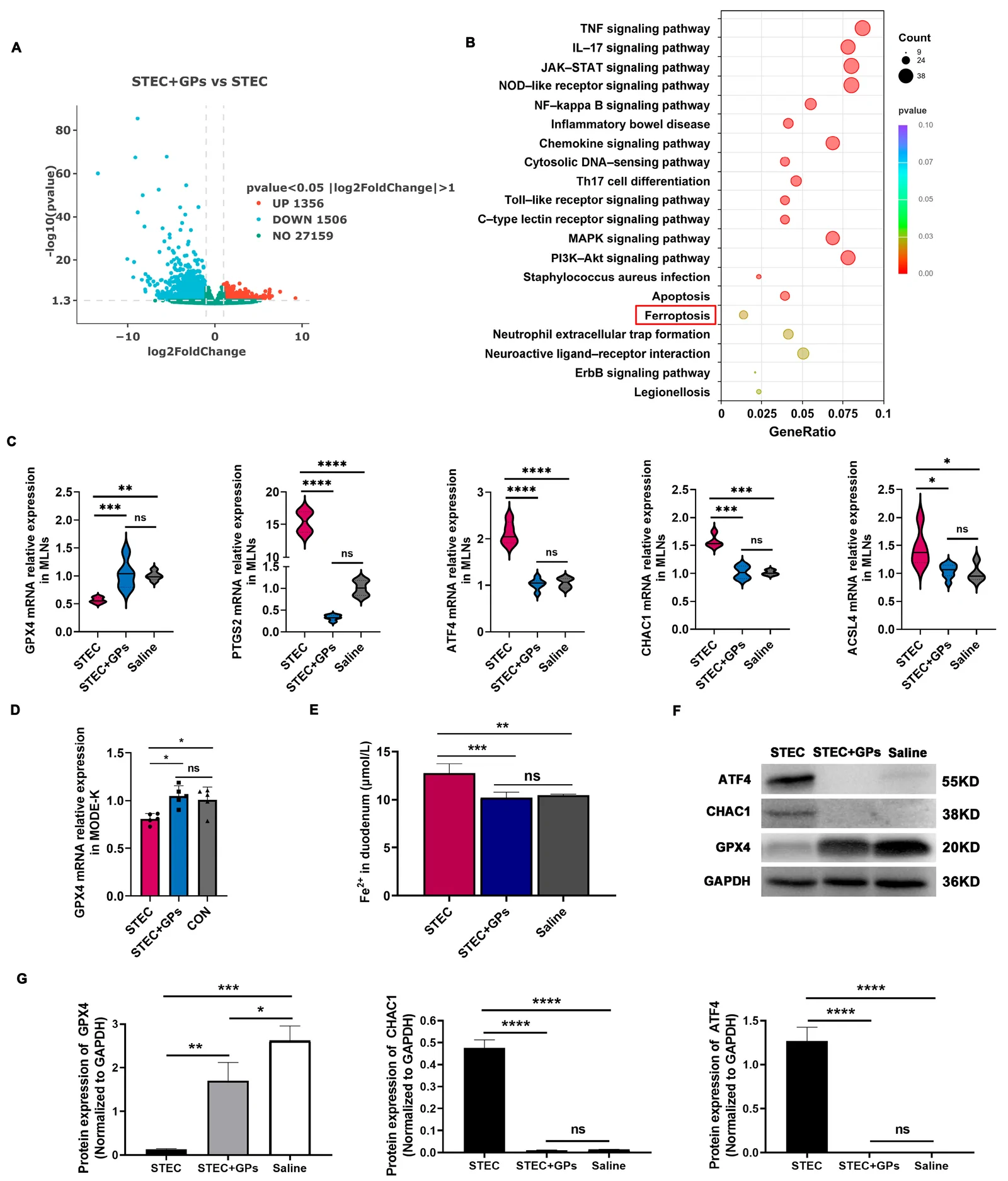
GPs attenuate STEC-induced ferroptosis. (**A**) Volcano plot of DEGs. (**B**) Significantly enriched KEGG pathways of downregulated genes. The red box highlights the ferroptosis pathway. (**C**) Expression changes in ferroptosis-related genes GPX4, PTGS2, ATF4, CHAC1 and ACSL4. (**D**) Validation of GPX4 expression in MODE-K cells. (**E**) Fe^2+^ levels in duodenal tissues. (**F**) Validation of ATF4, CHAC1, and GPX4 protein expression by Western blot. Western blot analysis was performed with three independent biological replicates, and representative blots are shown. All samples were processed in parallel under identical conditions. (**G**) Densitometric quantification of all Western blot replicates. Band intensities were normalized to GAPDH, and data are presented as mean ± SD (*n* = 3). * *p* < 0.05, ** *p* < 0.01, *** *p* < 0.001,**** *p* < 0.0001. ns, no significant difference.

To further elucidate the host protective mechanism of GPs against STEC-induced intestinal injury, transcriptome sequencing analysis was performed in MLNs. Transcriptomic analysis identified a total of 2862 DEGs, among which 1356 were significantly upregulated and 1506 were significantly downregulated (Figure 7A and Supplementary Table S2). To determine the biological functions of downregulated DEGs involved in responses to GP pretreatment, KEGG enrichment analysis was performed (Figure 7B and Supplementary Table S3). The KEGG pathways “TNF signaling pathway”, “IL-17 signaling pathway”, “NOD-like receptor signaling pathway”, “NF-κB signaling pathway”, “inflammatory bowel disease”, “Toll-like receptor signaling pathway”, “MAPK signaling pathway”, “apoptosis”, “ferroptosis”, and “neutrophil extracellular trap formation” were significantly suppressed. Notably, the significant suppression of the ferroptosis pathway suggests that GPs may exert protective effects against STEC in association with regulation of ferroptosis.

Ferroptosis is iron-dependent cell death driven by lipid peroxidation. Key regulators include GPX4, a critical antioxidant enzyme that inhibits ferroptosis, and PTGS2, ACSL4, ATF4, and CHAC1, which promote ferroptosis. PTGS2 and ACSL4 are positive markers, while GPX4 downregulation is a hallmark of ferroptosis. As shown in Figure 7C, compared with the saline group, STEC infection significantly suppressed GPX4 expression while upregulating PTGS2, ATF4, CHAC1, and ACSL4 expression. In contrast, GP pretreatment markedly restored GPX4 expression and reduced the expression of PTGS2, ATF4, CHAC1, and ACSL4 to levels comparable to those in the saline group, indicating that GPs effectively reverse STEC-induced molecular changes associated with ferroptosis. Furthermore, the changes in GPX4 expression were validated in vitro using mouse intestinal epithelial MODE-K cells (Figure 7D). Iron ions play a central role in ferroptosis. Excessive ferrous iron (Fe^2+^) catalyzes the generation of hydroxyl radicals via the Fenton reaction, thereby exacerbating lipid peroxidation. Measurement of Fe^2+^ levels in duodenal tissues revealed a significant elevation in the STEC group, whereas GP treatment significantly reduced iron accumulation to control levels (Figure 7E). These results suggest that STEC infection disrupts iron metabolism homeostasis, leading to iron overload, while GPs contribute to restoring iron homeostasis and alleviating iron overload, which may help counteract ferroptosis. Western blot analysis further confirmed the above findings. As shown in Figure 7F,G, STEC infection activated the protein expression of ATF4 and CHAC1 while downregulating GPX4. Conversely, GP treatment suppressed the expression of ATF4 and CHAC1 and restored GPX4 protein expression, further suggesting that GP-mediated regulation of the ATF4-CHAC1-GPX4 axis is associated with the suppression of ferroptosis.

## 4. Discussion

In the present study, we provide evidence that GPs confer protection against STEC-induced intestinal injury through a multi-target mechanism centered on the ATF4-CHAC1-GPX4 axis. Our findings reveal a previously unrecognized mechanistic link between STEC infection and this ATF4-CHAC1-GPX4-driven cell death program, positioning GPs as a novel, non-antibiotic protective agent for STEC-associated enteropathy.

A key limitation of this study is that i.p. injection bypasses the natural gastrointestinal route of STEC entry. Oral gavage in conventional mice failed to induce any phenotype even at 5 × 10^9^ CFU/mouse, consistent with the recognized difficulty of establishing STEC enteric infection without antibiotic preconditioning [27]. We recognize that this model does not recapitulate the intestinal colonization phase, and that systemic challenge may evoke responses that partly reflect sepsis rather than pure luminal enteropathy. We therefore recognize that this model does not replace natural oral infection, which would require further optimization in future studies. Additionally, the 7-day GP pretreatment regimen was prophylactic rather than therapeutic; future studies with post-infection GP administration will be needed to evaluate true therapeutic efficacy. Furthermore, the in vitro experiments were performed using a single intestinal epithelial cell line, MODE-K; validation in additional intestinal models such as IPEC-J2, Caco-2, or primary organoids would be required to confirm that the observed effects are not cell-line-specific. Using this model, we found that STEC infection typically leads to severe diarrheal symptoms and 100% mortality in susceptible mouse models within 108 h post-infection. In contrast, GP pretreatment significantly improved survival, with the 100 mg/kg dose achieving 75% survival at 24 h and 55% at 168 h. This survival benefit was accompanied by marked alleviation of diarrhea, reduced bacterial colonization and dissemination in systemic organs and feces, and diminished Stx2 levels in both the spleen and duodenum. Importantly, GPs exhibited no direct cytotoxicity against MODE-K cells across a wide concentration range, supporting their favorable safety profile. These findings support the concept that plant-derived saponins, unlike conventional antibiotics that trigger the SOS response, combat STEC primarily by attenuating virulence rather than through direct bactericidal activity. Notably, the pronounced reduction in Stx2 levels by GPs is particularly clinically relevant, as Shiga toxins are major drivers of systemic complications. By reducing toxin burden while preserving host cell viability, GPs may effectively interrupt the downstream pathological cascade without exacerbating toxin release via SOS-mediated phage induction.

Beyond direct anti-pathogenic effects, GPs markedly counteracted STEC-induced gut microbiota dysbiosis. STEC infection substantially reduced α-diversity and altered β-diversity, with a marked expansion of the pathogenic genus *Escherichia-Shigella*. It should be noted that 16S rRNA sequencing at the genus level cannot distinguish STEC from other *Escherichia-Shigella* strains; thus, the increased abundance of this genus should not be directly equated with STEC-specific expansion. GP pretreatment effectively reversed these changes, increasing microbial diversity and specifically enriching beneficial genera including *Lactobacillus* and *Odoribacter*. This prebiotic effect is consistent with prior reports on gypenosides and other plant polysaccharides [28,29]. The enrichment of *Lactobacillus* is particularly noteworthy, as *Lactobacilli* are known to inhibit STEC colonization through competitive exclusion, production of short-chain fatty acids, and enhancement of mucosal barrier function [8,30,31]. Given that gut microbiota dysbiosis compromises disease resistance via metabolic imbalance, barrier disruption, and immune dysregulation, the restoration of microbial ecology by GPs likely represents a critical contributor to the overall host defense against STEC.

In parallel, GPs exerted prominent immunomodulatory effects that may contribute to their antimicrobial activity. STEC infection induced significant systemic and local immune imbalances, characterized by reduced total white blood cell counts and lymphocyte percentages, elevated neutrophils and monocytes, and pronounced shifts in lymphocyte populations. In the spleen, STEC infection selectively reduced CD4^+^ T cells without affecting CD8^+^ T cells, NK cells, dendritic cells, or B cells, whereas in the MLNs, it caused broader disruptions, including decreases in both CD4^+^ and CD8^+^ T cells and NK cells, alongside an increase in B cells. These tissue-specific alterations suggest that STEC compromises systemic adaptive immunity while markedly disrupting local mucosal immune homeostasis. GP pretreatment effectively reversed these immunological perturbations, albeit with distinct tissue-specific patterns. In the spleen, GPs restored CD4^+^ T cell proportions to near-normal levels, while in the MLNs, they selectively recovered CD8^+^ T cells and NK cells without affecting CD4^+^ T cells. Notably, GPs elevated NK cells and dendritic cells in the MLNs above saline-control levels, suggesting an enhancement of mucosal immune surveillance. This tissue-divergent immunomodulation is functionally relevant: the restoration of splenic CD4^+^ T cells likely supports systemic antibody responses and pathogen clearance, whereas the enrichment of CD8^+^ T cells and NK cells in the MLNs may bolster local cytotoxicity against STEC-colonized epithelial cells. Concurrently, GPs effectively rebalanced the inflammatory milieu in both tissues, suppressing IL-1β, TNF-α, and IL-6 while restoring IL-10 gene expression. Given that excessive pro-inflammatory cytokines can exacerbate intestinal tissue damage and disrupt epithelial barrier integrity [32], this rebalancing of the inflammatory milieu likely contributes to the protective effects of GPs by limiting immunopathology and preserving gut barrier function. It should be noted that the recovery of CD4^+^ T cell proportions reflects phenotypic normalization rather than definitive functional restoration, which would require additional functional assays.

STEC infection caused severe disruption of tight junction proteins ZO-1 and Claudin-1, accompanied by villus rupture, shedding, and increased serum levels of DAO and D-LA, which are established biomarkers of intestinal barrier dysfunction. GP pretreatment restored tight junction continuity and ultrastructural integrity. Concurrently, we observed substantial oxidative stress in the duodenum of STEC-infected mice, evidenced by elevated ROS, MDA, and COX-2, along with depleted antioxidant enzymes including SOD, CAT, and GSH-Px, as well as a reduced GSH/GSSG ratio. Ultrastructural examination further revealed mitochondrial swelling, cristae disruption, and vacuolization, features suggestive of both ferroptosis and aberrant mitophagy. These mitochondrial abnormalities, together with the accompanying redox imbalance, prompted us to investigate whether STEC infection activates ferroptotic pathways and whether GPs confer protection through inhibition of this cell death program.

Collectively, the above findings support an integrated model in which GPs protect against STEC-induced intestinal injury involving coordination of two complementary arms. On one hand, GPs exert direct anti-pathogenic effects by attenuating Stx2 production and limiting bacterial dissemination, without exerting bactericidal pressure that would trigger SOS-mediated toxin release. On the other hand, GPs reinforce host defenses through three interconnected layers: restoring gut microbiota homeostasis, rebalancing systemic and mucosal immune responses, and preserving intestinal barrier integrity. These host-directed effects are functionally interconnected: microbiota restoration supports immune regulation, which limits inflammatory barrier damage, and barrier preservation restricts bacterial translocation and toxin dissemination, collectively amplifying the overall protective efficacy. This multi-target mechanism ultimately translates into improved survival and reduced intestinal pathology, positioning GPs as a promising non-antibiotic protective candidate for STEC-associated enteropathy.

Mechanistically, transcriptomic analysis of MLNs suggested that GP treatment was associated with suppression of the ferroptosis pathway, along with TNF, IL-17, NOD-like receptor, NF-κB, and MAPK signaling. Given its central role in the observed transcriptomic changes, we focused on the ferroptosis-associated ATF4-CHAC1 axis. STEC infection markedly upregulated ATF4 and its downstream target CHAC1, a glutathione-degrading enzyme, while concurrently downregulating GPX4 in both duodenal tissues and MODE-K cells. This molecular signature, characterized by GPX4 suppression accompanied by elevated ATF4 and CHAC1 expression, is a well-established hallmark of ferroptosis [33]. CHAC1 functions as a γ-glutamyl cyclotransferase that degrades glutathione, thereby influencing calcium signaling and mitochondrial function, and is recognized as a key regulator of oxidative stress and ferroptosis [34,35]. Its expression is transcriptionally controlled by factors including ATF4 and ATF3. Notably, the ATF4-CHAC1 axis has been shown to mediate ER stress-dependent ferroptosis through glutathione depletion and GPX4 suppression [35]. Under ER stress conditions, this transcriptional regulation proceeds via the PERK-eIF2α-ATF4 pathway, frequently in coordination with ATF3 and CHOP [36,37]. In line with this, we observed excessive iron accumulation, as indicated by elevated Fe^2+^ levels, alongside reduced glutathione availability, both conditions that favor lipid peroxidation. GP treatment effectively restored GPX4 expression, attenuated ATF4 and CHAC1 upregulation, and ameliorated iron overload, which may contribute to the suppression of ferroptosis. Western blot analysis further corroborated the reciprocal regulation of ATF4, CHAC1, and GPX4 at the protein level.

The ultrastructural evidence of mitochondrial vacuolization suggests that STEC also triggers excessive mitophagy, which can become maladaptive under severe oxidative stress [38]. ATF4 is known to transcriptionally regulate multiple autophagy genes through the eIF2α–ATF4 pathway, including Atg16l1, Map1lc3b, Atg12, Atg3, Beclin1, and Gabarapl2 [36]. Recent studies have demonstrated that ATF4 activation exacerbates mitochondrial membrane potential loss, reduces ATP synthesis, and promotes mitochondrial fragmentation while impairing mitophagic flux [36]. These observations suggest that GPs may help preserve mitochondrial integrity, potentially involving modulation of the ATF4-regulated autophagy pathway. However, specific markers for mitophagy were not assessed in this study, and thus the role of mitophagy in the observed mitochondrial protection remains speculative. The systemic improvement of oxidative stress markers was associated with coordinated changes in the ATF4-CHAC1-GPX4 axis following GP treatment. CHAC1-mediated glutathione degradation represents a critical bridge connecting ER stress to oxidative stress [39]. GPX4 prevents ferroptosis by reducing lipid hydroperoxide levels, with GSH serving as an essential substrate for this protective mechanism [34]. Depletion of GSH disrupts this defense, leading to lipid peroxide accumulation and ferroptotic cell death [40]. In our study, GP treatment correlated with maintained glutathione homeostasis and preserved GPX4 function, a pattern consistent with the known multi-target protective actions of plant-derived compounds. It should be noted that our mechanistic evidence is primarily correlative. While we observed consistent changes in Fe^2+^ levels and ATF4, CHAC1, and GPX4 expression following GP treatment, these data do not establish that GPs act directly through this pathway. Future studies employing genetic or pharmacological manipulations will be necessary to definitively establish causality. Future studies with post-infection GP administration are needed to evaluate true therapeutic efficacy.

## 5. Conclusions

Our data support a conceptual framework in which GP treatment is associated with modulation of the ATF4-CHAC1-GPX4 axis, alongside suppression of ferroptosis and preservation of mitochondrial integrity. This molecular intervention is synergistically complemented by gut microbiota and immune homeostasis restoration, which collectively reinforce intestinal barrier integrity. The convergence of these multi-level mechanisms may contribute to the in vivo suppression of STEC pathogenicity. Our findings provide a correlative mechanistic framework for developing novel anti-infective strategies against STEC infection.

## Data Availability

The original data presented in the study are openly available in the NCBI SRA database under the accession number PRJNA1499905 (https://www.ncbi.nlm.nih.gov/sra/PRJNA1499905, approved on 24 July 2026).

## Supplementary Materials

The following supporting information can be downloaded at https://www.mdpi.com/article/10.3390/antiox15091103/s1, Table S1: List of primers used for quantitative real-time PCR analysis; Table S2: Detailed information of differentially expressed genes (DEGs) between GPs-treated and non-GPs-treated STEC-infected mice; Table S3: The list of KEGG enrichment analysis of downregulated genes between GPs-treated and non-GPs-treated STEC-infected mice.
