# Supplementary material for: Gypenosides Mitigate STEC-Induced Intestinal Injury Through Modulation of Ferroptosis Involving the ATF4-CHAC1-GPX4 Axis
Source: Antioxidants (Basel). 2026 Sep 1;15(9):1103. doi: 10.3390/antiox15091103 (PMC13603104; doi:10.3390/antiox15091103)
Supplement: Supplementary file 1 [file antioxidants-15-01103-s001.zip › supplement Table S1.pdf]

Supplementary Table S1 List of primers used for quantitative real-time PCR analysis

Target	Mature sequence (5’ - 3’)
IL-10	F: GGGTTGCCAAGCCTTATCG
	R: GCTTCTCACCCAGGGAATTC
IL-1β	F: CTCACAAGCAGAGCACAAGC
	R: CAGTCCAGCCCATACTTTAGG
IL-6	F: TGACAATATGAATGTTGGGACA
	R: TTCCAAGAAACCATCTGGCTA
TNF-α	F: CTGTAGCCCACGTCGTAGC
	R: TTGAGATCCATGCCGTTG
Stx2	F: CGTCACTCACTGGTTTCATCAT
	R: TCTGTATCTGCCTGAAGCGTAA
GPX4	F: GCCTGGATAAGTACAGGGGTT
	R: CATGCAGATCGACTAGCTGAG
PTGS2	F: TGCACTATGGTTACAAAAGCTGG
	R: TCAGGAAGCTCCTTATTTCCCTT
ATF4	F: CATGGCGTATTAGAGGCAGC
	R: ACACTGCTGCTGGATTTCGT

CHAC1	F: TATAGTGACAGCCGTGTGGG
	R: GCTCCCCTCGAACTTGGTAT
ACSL4	F: ACTGGCGATATTGGAGAAT
	R: CACATAGGACTGGTCACTT
GAPDH	F: TGCACCACCAACTGCTTAG
	R: GGATGCAGGGATGATGTTC
